# Automatic detection and recognition of nasopharynx gross tumour volume (GTVnx) by deep learning for nasopharyngeal cancer radiotherapy through magnetic resonance imaging

**DOI:** 10.1186/s13014-023-02260-1

**Published:** 2023-05-08

**Authors:** Yandan Wang, Hehe Chen, Jie Lin, Shi Dong, Wenyi Zhang

**Affiliations:** 1 Faculty of Computer Science and Technology, Wenzhou University, WenZhou, China; 2 College of Intelligent Manufacturing, Wenzhou Polytechnic, Wenzhou, China; 3 Department of Radiology, The First Affiliated Hospital of Wenzhou Medical University, WenZhou, China; 4 Department of Radiotherapy, Wenzhou Central Hospital, Dingli Clinical Medical School of Wenzhou Medical University, Wenzhou, China; 5 Department of Radiotherapy, The First Affiliated Hospital of Wenzhou Medical University, WenZhou, China

**Keywords:** Deep learning, Automatic detection and recognition, Nasopharyngeal cancer, Magnetic resonance imaging, Target delineation

## Abstract

**Background:**

In this study, we propose the deep learning model-based framework to automatically delineate nasopharynx gross tumor volume (GTVnx) in MRI images.

**Methods:**

MRI images from 200 patients were collected for training-validation and testing set. Three popular deep learning models (FCN, U-Net, Deeplabv3) are proposed to automatically delineate GTVnx. FCN was the first and simplest fully convolutional model. U-Net was proposed specifically for medical image segmentation. In Deeplabv3, the proposed Atrous Spatial Pyramid Pooling (ASPP) block, and fully connected Conditional Random Field(CRF) may improve the detection of the small scattered distributed tumor parts due to its different scale of spatial pyramid layers. The three models are compared under same fair criteria, except the learning rate set for the U-Net. Two widely applied evaluation standards, mIoU and mPA, are employed for the detection result evaluation.

**Results:**

The extensive experiments show that the results of FCN and Deeplabv3 are promising as the benchmark of automatic nasopharyngeal cancer detection. Deeplabv3 performs best with the detection of mIoU 0.8529 ± 0.0017 and mPA 0.9103 ± 0.0039. FCN performs slightly worse in term of detection accuracy. However, both consume similar GPU memory and training time. U-Net performs obviously worst in both detection accuracy and memory consumption. Thus U-Net is not suggested for automatic GTVnx delineation.

**Conclusions:**

The proposed framework for automatic target delineation of GTVnx in nasopharynx bring us the desirable and promising results, which could not only be labor-saving, but also make the contour evaluation more objective. This preliminary results provide us with clear directions for further study.

**Supplementary Information:**

The online version contains supplementary material available at 10.1186/s13014-023-02260-1.

## Key points

• *The precise delineation of nasopharynx gross tumor volume (GTVnx) is a critical step for nasopharyngeal cancer radiotherapy.*
• *Three deep learning models were trained to automatically delineate GTVnx.*
• *Automatic detection and recognition of GTVnx based on those models revealed desirable results and brought a positive impact on improving delineation accuracy and reducing workload.*

**Supplementary Information:**

The online version contains supplementary material available at 10.1186/s13014-023-02260-1.

## Introduction

As reported by the International Agency for Research on Cancer, there were around 129,000 new cases of nasopharyngeal cancer diagnosed in 2018, which accounts for 0.7% of all types of cancers [1]. More than 70% of new cases are in east and southeast Asia, especially with an age­standardised rate (world) of 3.0 per 100,000 in China [2]. Radiotherapy is the mainstay in the treatment of nasopharyngeal cancer, with intensity-modulated radiotherapy (IMRT) having been a major breakthrough in its treatment [3]. Since the anatomical structure and notoriously narrow treatment boundaries are complex, the target delineation of nasopharyngeal cancer still remains challenging. Accurate target delineation is crucial to achieving optimal tumour control and sparing of critical organs. However, manual delineation of nasopharynx gross tumour volume (GTVnx) is labour-intensive and has low reproducibility.

Automatic delineation of radiotherapy target as well as critical organs based on deep learning models have become a hot research topic in recent years [4–7]. Following international guidelines [8] and the RTOG 0225 protocol [9], a contrast-enhanced MRI was fused with treatment plan CT images using computer optimization to accurately delineate the primary disease. Thus, a contrast-enhanced MRI is recommended as the gold-standard imaging for nasopharyngeal cancer delineation. MRI-guided manual GTVnx delineation is challenging and time consuming due to its complex anatomical structure. Moreover, inter-observer variability in tumour delineation is not negligible [10]. Therefore, automatic delineation based on deep learning models could be a desirable alternative to overcome these difficulties. The deep learning models can detect tumour invasion more accurately and assist junior oncologists in GTVnx delineation. Compared to the conventional method, the proposed method could not only save time but also make the contours evaluation more objective.

A few studies have applied deep learning-associated models to nasopharyngeal cancer. A deep learning based model was used to predict achievable dose-volume histograms(DVHs) of organs at risk(OARs) for automation of inverse planning in nasopharyngeal cancer [11]. A modified deep learning model called U-Net was used to automatically segment and delineate tumour targets in CT images in nasopharyngeal cancer [12]. In this paper, MRI was used for its better resolution when it comes to soft tissues, perineural and skull base invasion as compared to CT [13]. We trained three different deep learning models to delineate GTVnx in MRI images with thoroughly comparison and analysis.

In this paper, we proposed deep learning based framework with three popular network models (FCN [14], U-Net [15], Deeplabv3[16]) aimed at achieving high-level semantic feature extraction for nasopharyngeal cancer contouring. In our work, we collect the nasopharynx cancer MRI image dataset, and manually draw the macroscopic tumour by using the expert graphical image annotation tool “Labelme [18]”, which was open sourced by CSAIL lab of MIT. The tool allows us to manually delineate the macroscopic tumour in MRI image with class annotated as ground truth training data aiming at training the deep learning based models. We propose the framework with three popular deep learning models based to automatically contour the GTVnx. The models are fed with large high resolution of 512 × 512 sized MRI images which capture various slices images for each individual patient.

## Methods

### Data collection and GTVnx contours

We retrospectively collected 2088 enhanced T1-weighted turbo spin echo sequence MR images with an axial slice thickness of 5 millimetres sized of 512 × 512 from 200 patients treated at The First Affiliated Hospital of WenZhou Medical University between January 2020 and December 2021. All the image data was acquired from 3.0T MRI (GE signa HDxt, USA or Philips Achieva, Holland). The final diagnosis of nasopharyngeal cancer was proven by pathology and immunohistochemical analysis. The macroscopic GTVnx contours were manually delineated by radiation oncologists with more than 10 years of clinical experience. To guarantee delineation accuracy, all the GTVnx contours were reviewed and rectified by more senior radiation oncologists together with other two radiation experts. Once the entire cohort of images datasets (2088 images of 200 patients) had been manually drawn, we followed the 90%-10% split rules to randomly split the data as training-validation and testing set. To make the testing independent from the training dataset, the 90%-10% random split was performed over the 200 patients rather than the entire 2088 images. The set split over patients could avoid the images from the same patient appear in both train-validation and testing set, which will make the model overfit and result in false high delineation accuracy.

### The proposed ACNC framework

**Fig. 1:**
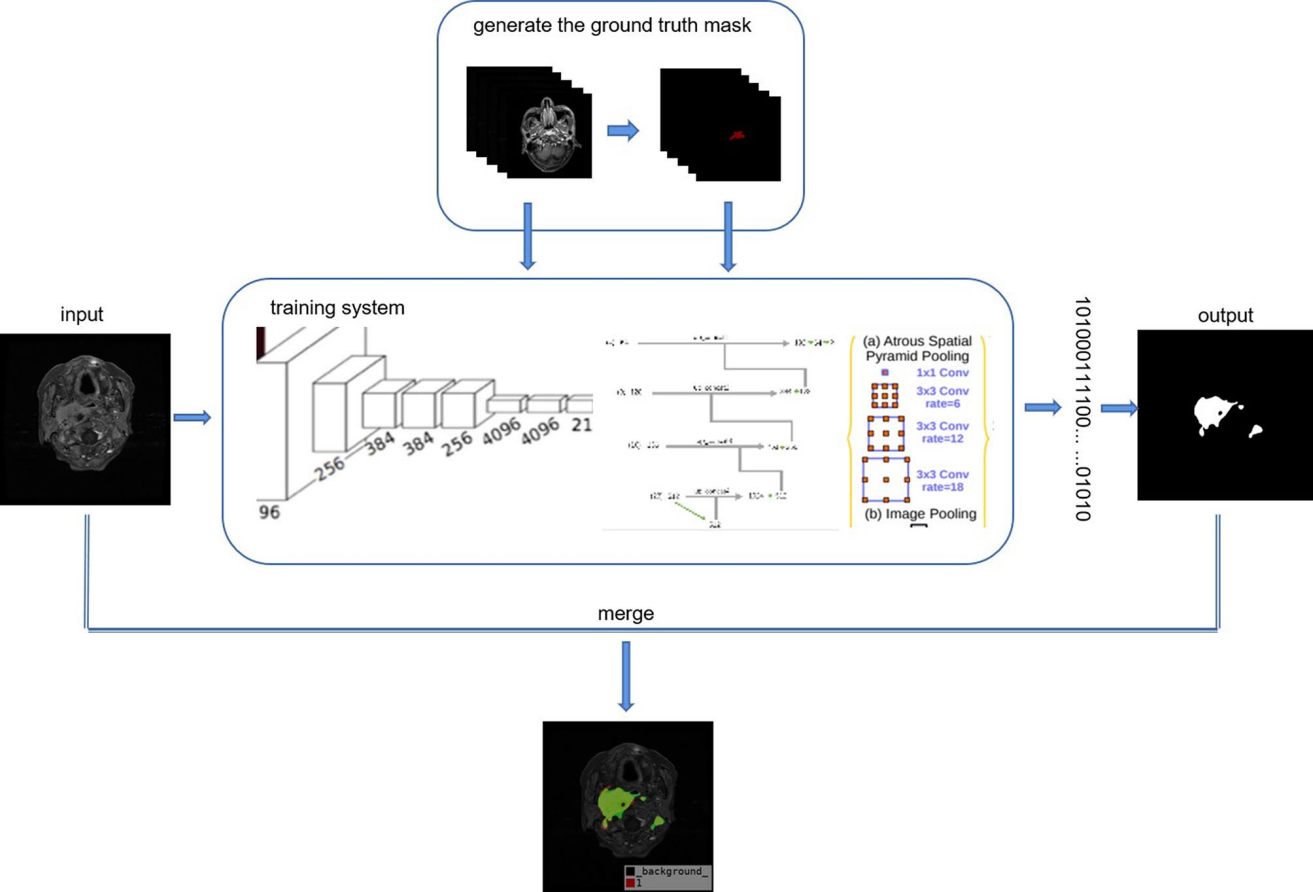
Automatic contour of nasopharynx cancer framework

Figure 1. shows the framework of our proposed Automatic Contour of Nasopharynx Cancer (ACNC). We first collect the nasopharynx cancer MRI dataset, and manually annotate the contour of tumour location generating ground truth mask images for each raw image. The raw images are then fed into the training system for some specified epochs in the guidance of the ground truth masks to search for the optimized weights solutions for the training system. Our training system include three deep neural networks, which are Fcn8s, U-Net and deeplabv3. Once the models are well trained with the 180 patients examples under the guidance of the corresponding ground truth, we then fed the trained system with the remaining 20 patients to test the performance of the trained system. As shown in Fig. 1. the raw image will pass the trained networks, the system will classify each pixel into binary that “1” denotes the tumour and “0” denotes the background. Finally, the one-dimensional binary classification result array will be reshaped to the original size of the raw input image producing the predicted tumour contour locations. To observe the prediction results better visually, we merge the raw image, ground truth mask image and the predicted result into one with ground truth in red and predicted result in green.

### FCN8s

Fully Convolutional Network (FCN)[14] was proposed for semantic image segmentation in 2015. Compared to traditional Convolution Neural Network (CNN), the fully convolutional network replaces the fully connection layers with the convolutional layers. Our backbone is using the vgg19 architecture that starts with two blocks consisting of two convolution layers with one Maxpooling operation, then followed by three blocks consisting of the four convolution layers with one Maxpooling operation. All the convolutional outputs are activated by the ReLU activation function. The network architecture is very similar to the one shown in Supplementary Fig. S1, the vgg16 architecture.

### U-Net architecture

**Fig. 2:**
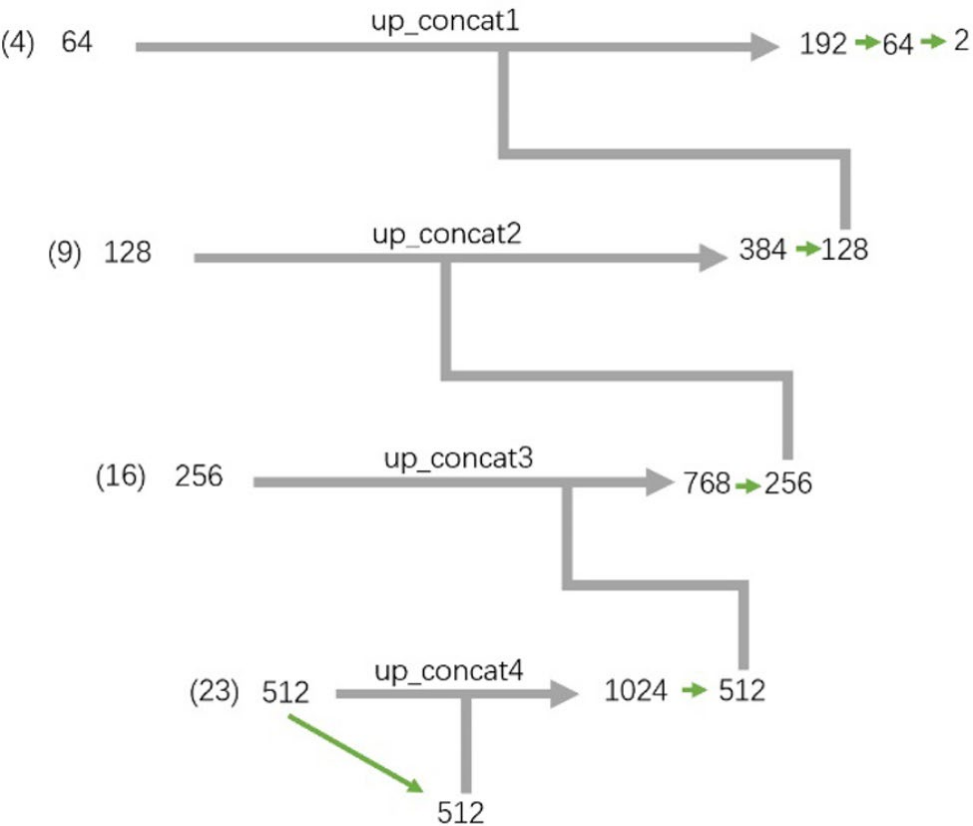
The details of skip connection for concatenation of U-Net

U-Net [15], mainly for biomedical image segmentation, was proposed in 2015 right two months after FCN proposed. To date, It has been widely applied for biomedical image segmentation due to its simplicity and superior performance. U-Net is well-known by its U-like network architecture, which is the process of encoding on the left half part of “U” and decoding on the right half part of “U”. The left half of “U” is used to extract the feature details of our nasopharyngeal tumour images, and the right half of “U” with skip connection of left part feature maps is used to recover the location information of each pixel. Then output the tumour contoured image with the same size as the original’s. Supplementary Fig. S1 shows the details architecture of the left down sampling “U”-Net which applies vgg16 as the backbone. The backbone consists two types of repeating blocks that are two convolutional layers followed by one max pooling layers and three convolutional layers followed by one max pooling layers respectively. The details of the kernel size, stride and padding information please refer to Supplementary Fig. S1. The detail of up sampling on the right part of U-Net is shown in Supplementary Fig. S2. To show how the Maxpool results are contracted as the up-sampling path, we present it in Supplementary Fig. S1. As shown in Supplementary Fig. S2, the number in braces is the feature layer derived from Supplementary Fig. S1. For example (4) and (9) as shown in Fig. 2. are derived by the repeating blocks of two convolutional layers and ReLU layers, then followed by one Maxpool. 64 and 128 beside to (4) and (9) are the outputs channels after passing the repeats blocks. The arrow path derives total channels by the concatenation of the channels from two paths. Furthermore, the features from left path will be randomly cropped for features augmentation purpose.

### DeepLabv3

Deeplabv3[16] was proposed for semantic image segmentation in 2017. It was improved based on the former two versions, we will brief the former two versions first to introduce the general knowledge of the Deeplab architecture, then highlight the key improvements of the third version. Deeplabv1 was derived by improving the DCNN by combining the responses at the final DCNN layer with a fully connected Conditional Random Field (CRF) to solve the poor localization problem existing in DCNN. In Deeplabv2, the backbone of VGG-16 was replaced by ResNet. Besides, Atrous Spatial Pyramid Pooling (ASPP) was proposed to solve the problem of detecting the object with various scales. Supplementary Fig. S3 and Supplementary Fig. S4 show the ASPP blocks of Deeplabv2 and Deeplabv3 respectively. From those figures, we can clearly see the improvements of ASPP proposed in Deeplabv3. In Deeplabv2, the ASPP block consists of four same sized 3 × 3 conv2d. Whilst in Deeplabv3, it starts with the block of one Conv2d sized at 3 × 3 stacked up by BN and RELU, then followed by another three blocks with same sized 3 × 3 Conv3d attached by BN and ReLU as well, finally it ends up with the block of AdaptiveAvg Pool2d, stack up with Conv2d, BN, ReLU and Bilinear Interpolate. Since ASPP is the main improvement of Deeplabv3, the rest of details please refer to the paper of interest. However, in our experiment, we use resnet101 to replace the backbone of Xception mentioned in paper [16].

## Results

**Fig. 3:**
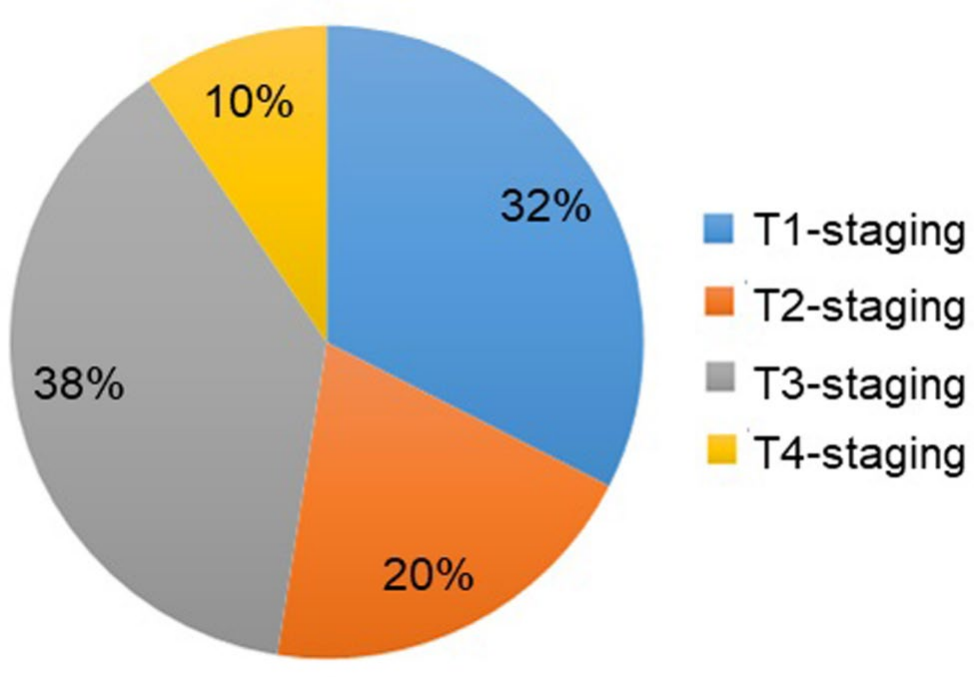
T-staging diagnosis results of nasopharyngeal carcinoma

**Fig. 4:**
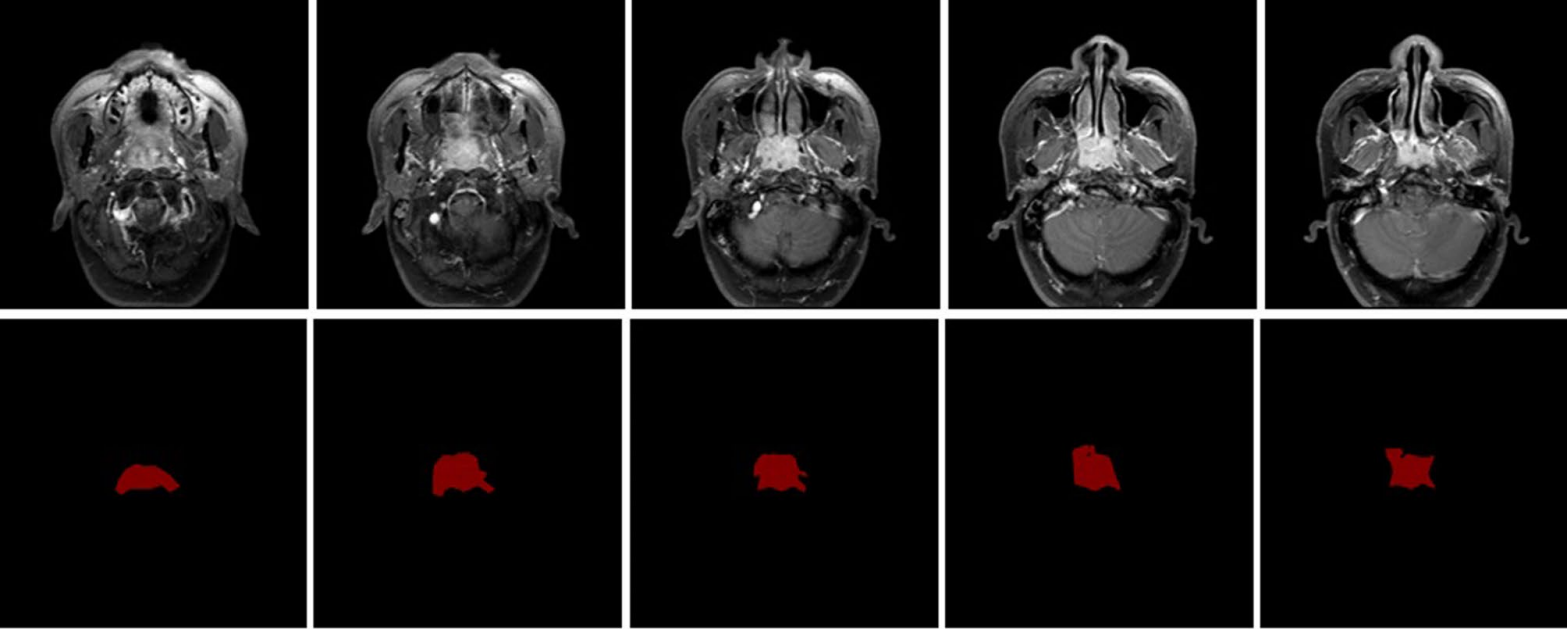
The samples of raw data and ground truth mask generated by “labelme” tools

The dataset from 200 nasopharyngeal cancer patients was reviewed. Those patients were scanned by GE signa HDxt 3.0T and achieva TX 3.0T MRI machines in total of 2088 MRI images with resolution of 512 × 512. Figure 3 indicates that, among the 200 patients, 65 cases of T1-staging, 39 cases of T2-staging, 76 cases of T3-staging, and 20 cases of T4-staging were diagnosed by routine MRI. Following the 90%-10% split rules, 180 patients that 1881 images are split as training-validation data and the rest 20 patient cases that 207 images are split as the testing data, following a widely used methodology [17]. Figure 4. shows our raw data examples and their corresponding ground truth contour masks drawn by our expert doctor with the expert annotation tool “Labelme” [18]. Besides, we list the detail of gender and age distribution of collected dataset in Table 1. From the table, we can clearly see that the number of male patients is more than three times bigger than the one of females. Moreover, the patients are mostly distributed between the age of 40 and 60, then followed by the age more than 60. In table, we also list the gender and age distribution in our randomly 90%-10% split training-validation and testing cohort. Please refer to the table for the details.

**Table 1:** The gender and age distribution of collected dataset

	Entire cohort	Training-Validation cohort	Testing cohort
Male	156	144	12
Female	44	36	8
Age (years)			
≤ 40	27	26	1
> 40 & ≤60	110	99	11
> 60	63	55	8

Our 1881 training images are also follow 90%-10% split rules that 90% are split as training data and the rest goes for the validation data. The purpose of the validation data is to avoid model overfitting that might occur during the early training phase,. We set the batch size to be 9, the learning rate to be 10^− 3^ for the FCN and Deeplabv3 model, 10^− 5^ for the U-Net. 200 epochs are set for the training circles, and 209 iterations will be passed for each epoch. Algorithm 1 gives the clear steps of the training, validation and testing process in details. We conduct our experiment on ubuntu 18.04 operating system with two TitanXp GPUs running in parallel mode. The FCN and Deeplabv3 model consumes only one GPU with 12 g memory. However, U-Net consumes much more memories, the two GPUs running in parallel are required.

**Algorithm 1:** Training, validation and testing steps

1: Read training, validation, testing, and mask set, process them into tensor 2: For epoch from 1 to 200 3: For iter_training_ from 1 to training_size_/batch_size_
4: pass the training data to the training system (FCN, Deeplabv3, U-Net), getting the train_result_ 5: accumulate the loss_train_ by the loss function between train_result_ and ground truth mask 6: update weights W_training_ 7: End iter training 8: For iter_val_ from 1 to val_size_/batch_size_
9: pass the validation data to the trained system, getting val_result_ 10: accumulate loss_val_ by the loss function between val_result_ and ground truth mask 11: End iter validation
12: print the average loss by loss_train_/(epoch + 1) and loss_val_/(epoch + 1)
13: End training and val 14: load the model with trained W_weights_ 15: pass testing data to the model and get test_result_
16: compute the mIoU, and mPA 17: merge the images of raw data with test_result_ and ground truth

The experiment results are evaluated in mean Intersection over Union (mIoU) and mean Pixel Accuracy (mPA) system. mIoU, as shown in formula 1, is widely used in image segmentation to evaluate the segmentation result that shows the mean intersection over union between the predicted result and the ground truth mask image. The mPA, as shown in formula 2, is the simplest way to evaluate the result by computing the average correct classified pixels over all pixels within each class.

1 $$mIoU=\frac{1}{n+1}\sum _{i=0}^{n}\frac{{p}_{ii}}{{\sum }_{j=0}^{n}{p}_{ij}+{\sum }_{j=0}^{n}{p}_{ji}-{p}_{ii}}$$

2 $$mPA=\frac{1}{n+1}\sum _{i=0}^{n}\frac{{p}_{ii}}{{\sum }_{j=0}^{n}{p}_{ij}}$$

**Fig. 5:**
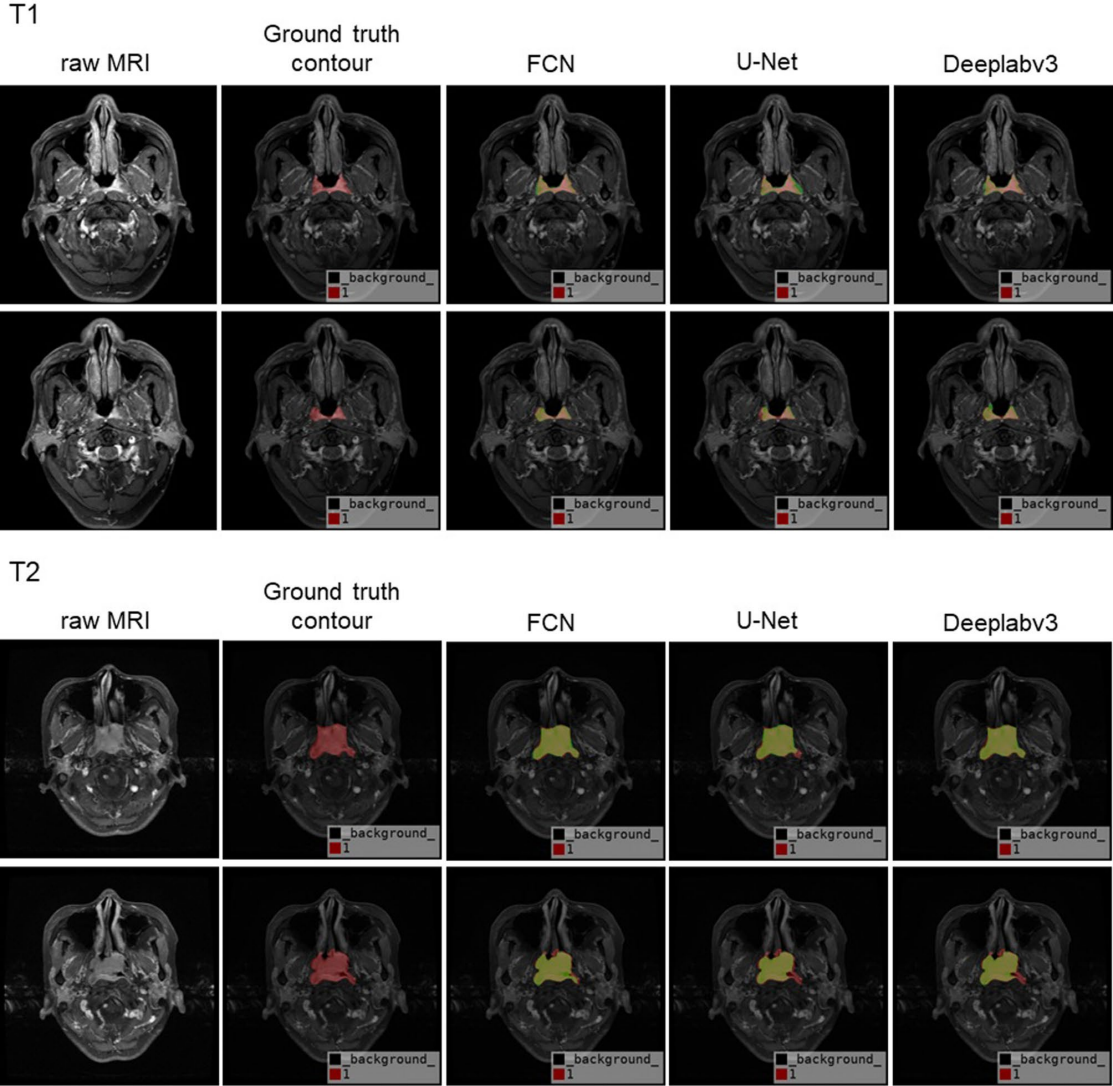
The automatic contour result (green) of T1 and T2-staging samples by using FCN, U-Net and Deeplabv3 respectively

**Fig. 6:**
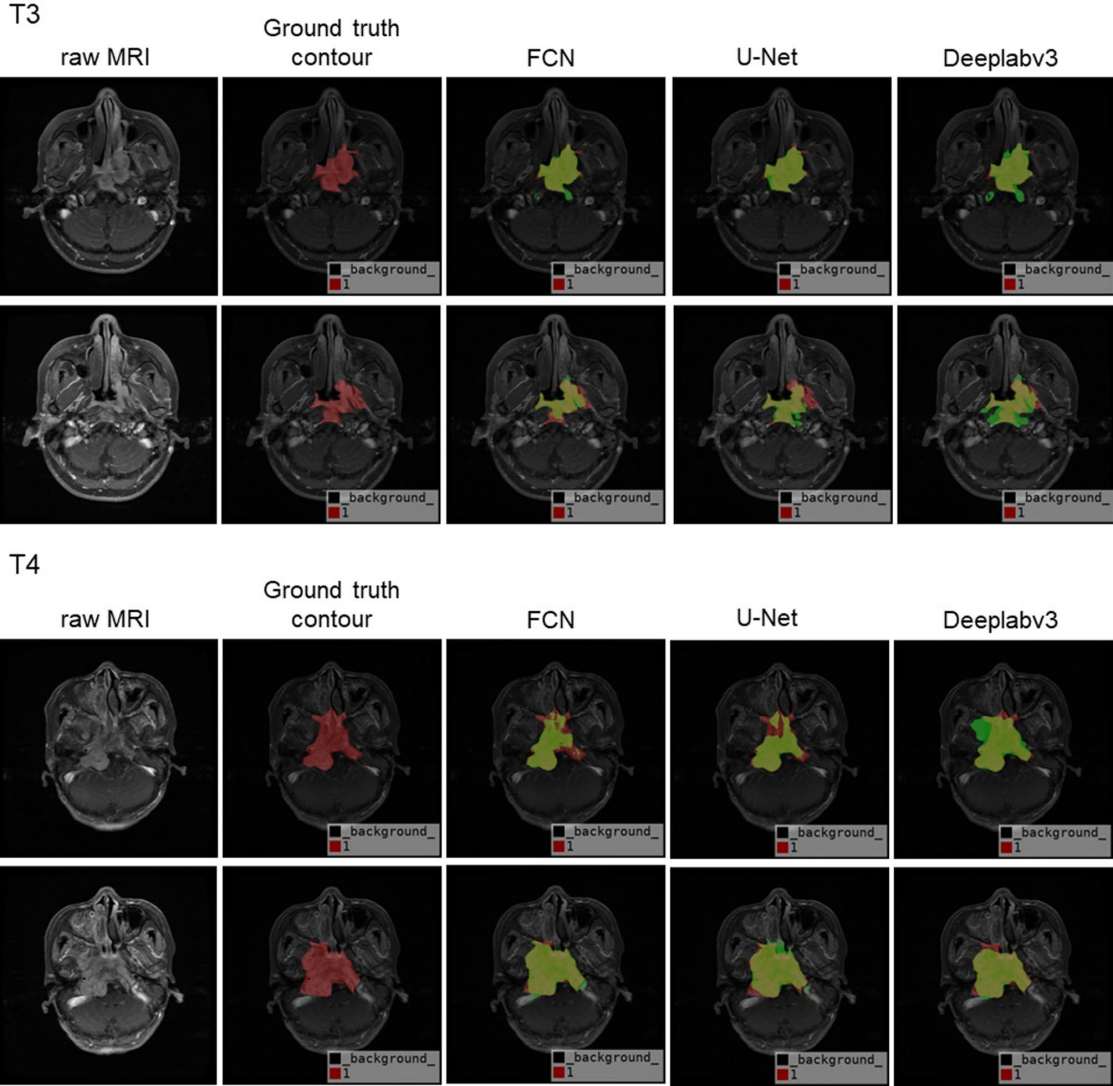
The automatic contour result (green) of T3 and T4-staging samples by using FCN, U-Net and Deeplabv3 respectively

From Table 2, we can clearly see our experiment results based on FCN, Deeplabv3 and U-Net model. Deeplabv3 performs best in both mIoU and mPA evaluation criteria with 0.8529 and 0.9103 mean results respectively over repeating experiments five times. Although FCN performs better than that of U-Net with mean 0.8497 of mIoU and 0.8936 of mPA respectively, it also gets higher result deviations with the deviation of mPA approaching to 0.0088, which is more than two times compared to that of U-Net and Deeplabv3. Besides, in the term of GPU memory consuming, U-Net consumes almost twice of that of FCN and Deeplabv3. Overall, Deeplabv3 is suggested in terms of recognition accuracy and GPU memory consumptions. Meanwhile, it also presents stable results with deviation considered. Visually, we present the automatic contour results of T1-staging, T2-staging, T3-staging, T4-staging in Figs. 5 and 6 respectively, by using the models of FCN, U-Net, and Deeplabv3. Each staging, we randomly outline four contour samples coming from the same patient in different slices. The first column layouts four raw MRI, followed by corresponding ground truth contour (red) in second column, the last three columns present the corresponding automatic contour (green) by using the models of FCN, U-Net, Deeplabv3 respectively. The results encourage us to look into this work further.

**Table 2:** The experiment result

Models	mIoU	mPA
FCN	0.8497$\pm$0.0040	$0.8936\pm$0.0088
U-Net	0.8272$\pm$0.0031	0.8770$\pm$0.0038
Deeplabv3	0.8529$\pm 0.0017$	0.9103$\pm$0.0039

## Discussion

In our study, the automatically contoured primary lesion of nasopharyngeal cancer were firstly performed by the three popular deep learning models (U-Net, Deeplabv3, FCN8s). We included 200 patient cases as the contouring dataset. DeepLabv3 was the best among the three deep learning models due to its ASPP block which is able to detect the target at various pyramid scales. For a fair comparison across the 3 models, we ensured regular parameters consistency, such as same epoch, learning rate, and batch size etc. However, we found that the learning rate of 10^− 3^ was completely not working for U-Net, while working well with Deeplabv3 and FCN8s. In U-Net model, the learning rate of 10^− 5^ performs almost 40% better than that of 10^− 3^ with the rest of parameters remain no changes. The result in Table 2 of U-Net comes from the learning rate of 10^− 5^. Theoretically, the larger learning rate could make the training converge earlier. However, practically, in our observation of the training and validation loss reached to the value of thousands at first training epoch. When the smaller learning rate of 10^− 5^ was applied to the U-Net model with the rest parameters not touched, the training and validation loss dropped rapidly to less than the value of one. The testing data passed to the trained model also generated the promising result as shown in Figs. 5 and 6 of the U-Net part. Besides, in our practical training experiment, U-Net also feedback us the GPU out of memory problem. The problem was solved by training the U-Net on two NVIDIA TitanXp 12 g GPUs in parallel. The reason why U-Net consumes much more memories could attribute to more weights from encoding phase are joined during the decoding phase when the concatenation operation happens. In the price of the memory consumption dramatically increased, U-Net did not bring us improved contour accuracy compared to the simplest FCN. Thus, practically, we conclude that U-Net is not suitable for the nasopharyngeal tumour delineation in spite of the model is classical and specifically proposed for the medical image segmentation. Though FCN is simple that stacked up with full convolutional layers only, it performs much better than U-Net in terms of both accuracy and memory consumption. Among the three models, although Deeplabv3 performs best, FCN is favoured considering the aspect of the complexity of the network architecture. We would like to further explore deeper in FCN architecture in our future work to seek the possibility to enhance the contour performance while remains the network architecture as simple as FCN does.

As it is known that deep learning models highly rely on training data, since they are big data driven models. Thus analysing on data samples will also significantly impact the performance of the models. From Table 1, we can see the obvious imbalance lined in collected data of gender and ages. The imbalanced data will result in unfair cohort performance analysis that make the analysis invalid. However, from the category data, it roughly made us pond over the following problems,

- Is nasopharyngeal cancer more common among men?
- Does nasopharyngeal cancer incidence peak between 40 and 60?

However, the two questions are derived from the categorized cohort data over 200 patients. The small size of the collected data may not be enough to answer the above two questions. We then learned the answers from the further reading key statistics of American Cancer Society reported that there are as many as 25 to 30 nasopharyngeal cancer cases per 100,000 men and 15 to 20 cases per 100,000 women in some parts of China. Meanwhile, the peak age of people being diagnosed is typically between 45 and 59. The key statistics for Nasopharyngeal Cancer of large-scale data samples ensures us the above-mentioned two questions.

The above two statistics also ensure us our further expansion of studies by employing the automatic deep learning based contour models to reveal the relationship between the complexity of the tumour structures (contours) and the biased genders/ages. As such, we could enhance the automatic delineation performance via more targeted data samples.

## Conclusion

In this paper, we proposed the deep learning model based framework to automatically draw the contours of the nasopharynx tumour in MRI images. In our framework, we proposed the three classic and popular deep learning models to evaluate the effectiveness of the deep learning model performing on the automatic detecting the nasopharynx tumour contours in MRI images. From the experiment results, Deeplabv3 performs better than the remaining two models. However, either from the precision results or visual results, there are still many cases in testing data not able to be well contoured, we attribute the problem to the lack of training data and the imbalance of the data distribution. To make our framework practical enough to assist radiation oncologists in saving time and making decisions, the promising preliminary results provide us with a clear clue for the further deeper study expansion.

In future work, we will keep collecting data on a more heterogenous and balanced patient cohort to improve model robustness. We will also try to design and improve the network architecture by analysing the reasons on the poor testing results derived by the automatic delineation proposed in this paper.

## Electronic supplementary material

Below is the link to the electronic supplementary material.

Supplementary Material 1

## Data Availability

Datasets used and/or analyzed during the current study are available from the corresponding author on reasonable request.

## Abbreviations

ACNC: Automatic Contour of Nasopharynx Cancer
ASPP: Atrous Spatial Pyramid Pooling
CNN: Convolution Neural Network
CRF: Conditional Random Field
DVHs: dose-volume histograms
FCN: Fully Convolutional Network
GTVnx: nasopharynx Gross Tumour Volume
IMRT: Intensity-Modulated Radiotherapy
OARs: Organs At Risk
mIoU: mean Intersection over Union
mPA: mean Pixel Accuracy
