# Supplementary material for: Automatic detection and recognition of nasopharynx gross tumour volume (GTVnx) by deep learning for nasopharyngeal cancer radiotherapy through magnetic resonance imaging
Source: Radiat Oncol. 2023 May 8;18:76. doi: 10.1186/s13014-023-02260-1 (PMC10165804; doi:10.1186/s13014-023-02260-1)

Supplementary Figure S1

```
(vgg): VGG(
  (features): Sequential(
    (0): Conv2d(3, 64, kernel_size=(3, 3), stride=(1, 1), padding=(1, 1))
    (1): ReLU(inplace=True)
    (2): Conv2d(64, 64, kernel_size=(3, 3), stride=(1, 1), padding=(1, 1))
    (3): ReLU(inplace=True)
    (4): MaxPool2d(kernel_size=2, stride=2, padding=0, dilation=1, ceil_mode=False)
    (5): Conv2d(64, 128, kernel_size=(3, 3), stride=(1, 1), padding=(1, 1))
    (6): ReLU(inplace=True)
    (7): Conv2d(128, 128, kernel_size=(3, 3), stride=(1, 1), padding=(1, 1))
    (8): ReLU(inplace=True)
    (9): MaxPool2d(kernel_size=2, stride=2, padding=0, dilation=1, ceil_mode=False)
    (10): Conv2d(128, 256, kernel_size=(3, 3), stride=(1, 1), padding=(1, 1))
    (11): ReLU(inplace=True)
    (12): Conv2d(256, 256, kernel_size=(3, 3), stride=(1, 1), padding=(1, 1))
    (13): ReLU(inplace=True)
    (14): Conv2d(256, 256, kernel_size=(3, 3), stride=(1, 1), padding=(1, 1))
    (15): ReLU(inplace=True)
    (16): MaxPool2d(kernel_size=2, stride=2, padding=0, dilation=1, ceil_mode=False)
    (17): Conv2d(256, 512, kernel_size=(3, 3), stride=(1, 1), padding=(1, 1))
    (18): ReLU(inplace=True)
    (19): Conv2d(512, 512, kernel_size=(3, 3), stride=(1, 1), padding=(1, 1))
    (20): ReLU(inplace=True)
    (21): Conv2d(512, 512, kernel_size=(3, 3), stride=(1, 1), padding=(1, 1))
    (22): ReLU(inplace=True)
    (23): MaxPool2d(kernel_size=2, stride=2, padding=0, dilation=1, ceil_mode=False)
    (24): Conv2d(512, 512, kernel_size=(3, 3), stride=(1, 1), padding=(1, 1))
    (25): ReLU(inplace=True)
    (26): Conv2d(512, 512, kernel_size=(3, 3), stride=(1, 1), padding=(1, 1))
    (27): ReLU(inplace=True)
    (28): Conv2d(512, 512, kernel_size=(3, 3), stride=(1, 1), padding=(1, 1))
    (29): ReLU(inplace=True)
    (30): MaxPool2d(kernel_size=2, stride=2, padding=0, dilation=1, ceil_mode=False)
  )
```

Supplementary Figure S2

```
    (up_concat4): unetUp(
      (conv1): Conv2d(1024, 512, kernel_size=(3, 3), stride=(1, 1), padding=(1, 1))
      (conv2): Conv2d(512, 512, kernel_size=(3, 3), stride=(1, 1), padding=(1, 1))
      (up): UpsamplingBilinear2d(scale_factor=2.0, mode=bilinear)
    )
    (up_concat3): unetUp(
      (conv1): Conv2d(768, 256, kernel_size=(3, 3), stride=(1, 1), padding=(1, 1))
      (conv2): Conv2d(256, 256, kernel_size=(3, 3), stride=(1, 1), padding=(1, 1))
      (up): UpsamplingBilinear2d(scale_factor=2.0, mode=bilinear)
    )
    (up_concat2): unetUp(
      (conv1): Conv2d(384, 128, kernel_size=(3, 3), stride=(1, 1), padding=(1, 1))
      (conv2): Conv2d(128, 128, kernel_size=(3, 3), stride=(1, 1), padding=(1, 1))
      (up): UpsamplingBilinear2d(scale_factor=2.0, mode=bilinear)
    )
    (up_concat1): unetUp(
      (conv1): Conv2d(192, 64, kernel_size=(3, 3), stride=(1, 1), padding=(1, 1))
      (conv2): Conv2d(64, 64, kernel_size=(3, 3), stride=(1, 1), padding=(1, 1))
      (up): UpsamplingBilinear2d(scale_factor=2.0, mode=bilinear)
    )
    (final): Conv2d(64, 2, kernel_size=(1, 1), stride=(1, 1))
  )
```

Supplementary Figure S3

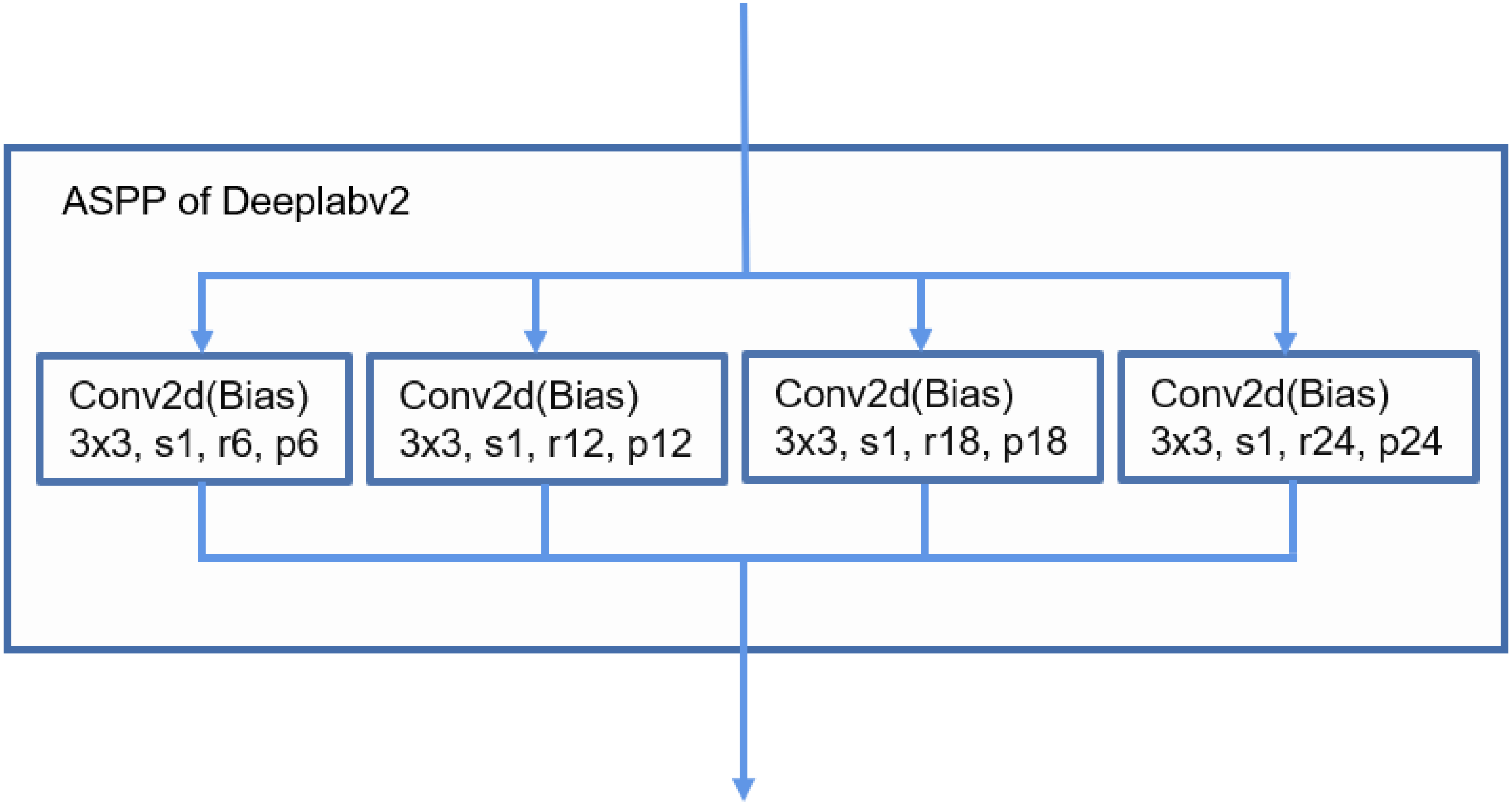

Supplementary Figure S4

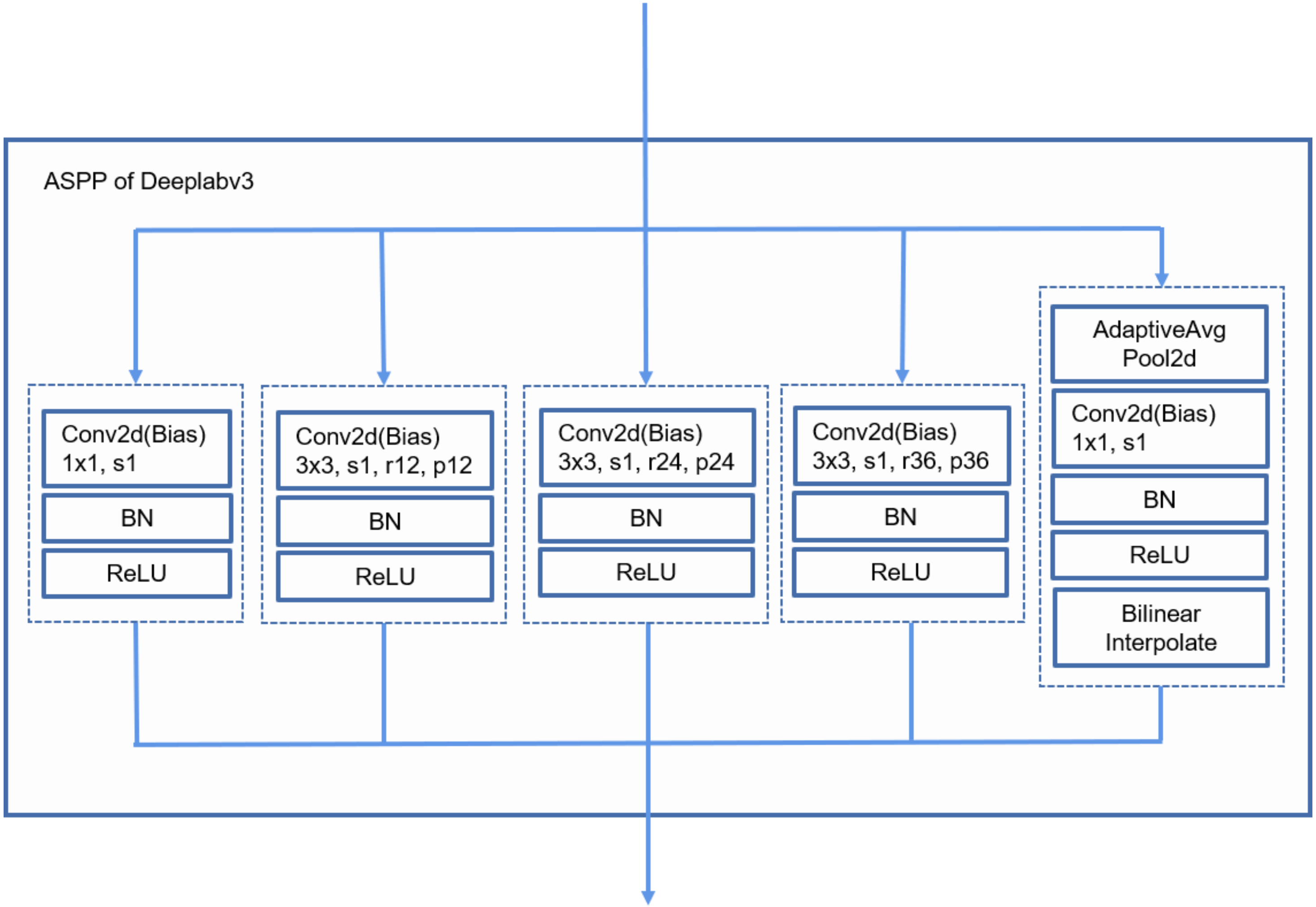

Supplement: Supplementary file 1 — Supplementary Material 1 [file 13014_2023_2260_MOESM1_ESM.pdf]
